# Identifying the neurodevelopmental and psychiatric signatures of genomic disorders associated with intellectual disability: a machine learning approach

**DOI:** 10.1186/s13229-023-00549-2

**Published:** 2023-05-23

**Authors:** Nicholas Donnelly, Adam Cunningham, Sergio Marco Salas, Matthew Bracher-Smith, Samuel Chawner, Jan Stochl, Tamsin Ford, F. Lucy Raymond, Valentina Escott-Price, Marianne B. M. van den Bree

**Affiliations:** 1 Centre for Academic Mental Health, Population Health Sciences, University of Bristol, Bristol, UK; 2 MRC Integrative Epidemiology Unit, Population Health Sciences, Bristol Medical School, University of Bristol, Bristol, UK; 3 Division of Psychological Medicine and Clinical Neurosciences, Centre for Neuropsychiatric Genetics and Genomics, Institute of Psychological Medicine and Clinical Neurosciences, Cardiff University School of Medicine, Hadyn Ellis Building, Maindy Road, Cathays, Cardiff, CF24 4HQ UK; 4 Department of Psychiatry, University of Cambridge, Cambridge, UK; 5 Department of Kinanthropology, Charles University, Prague, Czechia; 6 Department of Medical Genetics, University of Cambridge, Cambridge, UK

**Keywords:** Intellectual disability, Genetic syndromes, Machine learning, Behavioural phenotypes

## Abstract

**Background:**

Genomic conditions can be associated with developmental delay, intellectual disability, autism spectrum disorder, and physical and mental health symptoms. They are individually rare and highly variable in presentation, which limits the use of standard clinical guidelines for diagnosis and treatment. A simple screening tool to identify young people with genomic conditions associated with neurodevelopmental disorders (ND-GCs) who could benefit from further support would be of considerable value. We used machine learning approaches to address this question.

**Method:**

A total of 493 individuals were included: 389 with a ND-GC, mean age = 9.01, 66% male) and 104 siblings without known genomic conditions (controls, mean age = 10.23, 53% male). Primary carers completed assessments of behavioural, neurodevelopmental and psychiatric symptoms and physical health and development. Machine learning techniques (penalised logistic regression, random forests, support vector machines and artificial neural networks) were used to develop classifiers of ND-GC status and identified limited sets of variables that gave the best classification performance. Exploratory graph analysis was used to understand associations within the final variable set.

**Results:**

All machine learning methods identified variable sets giving high classification accuracy (AUROC between 0.883 and 0.915). We identified a subset of 30 variables best discriminating between individuals with ND-GCs and controls which formed 5 dimensions: conduct, separation anxiety, situational anxiety, communication and motor development.

**Limitations:**

This study used cross-sectional data from a cohort study which was imbalanced with respect to ND-GC status. Our model requires validation in independent datasets and with longitudinal follow-up data for validation before clinical application.

**Conclusions:**

In this study, we developed models that identified a compact set of psychiatric and physical health measures that differentiate individuals with a ND-GC from controls and highlight higher-order structure within these measures. This work is a step towards developing a screening instrument to identify young people with ND-GCs who might benefit from further specialist assessment.

**Supplementary Information:**

The online version contains supplementary material available at 10.1186/s13229-023-00549-2.

## Background

Up to 20% of individuals with a neurodevelopmental disorder have an identifiable genomic condition [1–4]. Such conditions include copy number variants, single nucleotide variants and aneuploidies, which we collectively call neurodevelopmental genomic conditions (ND-GCs). ND-GCs have been associated with schizophrenia [5], attention deficit hyperactivity disorder (ADHD), autism spectrum disorder (ASD) [6], and intellectual disability (ID) [7].

The clinical presentation of ND-GCs is variable and complex. For example, children with 22q11.2 deletion syndrome, a disorder caused by a deletion in the q11 region of chromosome 22, have a high risk of developmental delay and intellectual disability [8], seizures (57%) [9], motor coordination problems (81%) [10], sleep disturbances (60%) [11] and psychiatric disorders [12]. Such complex presentation is not unique to 22q11.2 deletion but is typical for many ND-GCs [13], as is incomplete and variable penetrance [14, 15].

It is therefore extremely important for families of a child with an ND-GC to be informed about the impact that the variant may have on their child’s development, so that they can obtain the best possible support. Additionally, clinicians, such as psychiatrists in child and adolescent mental health, or community learning disability services, who care for affected children after they have received a genetic diagnosis are challenged by complex presentations where symptoms which may require input from multiple clinical specialities are present.

This problem can be exacerbated by variability in the conditions that present in children with a ND-GCs, which may not follow the expected symptom patterns based on research from non-genotyped populations. For example, we have observed that children with 22q11.2 deletion and ADHD are much more likely to be affected with an inattentive subtype than the children with idiopathic ADHD [16]. A clinician who is unaware of this may be less likely to diagnose ADHD, meaning that the child misses beneficial treatment. Diagnostic overshadowing may also take place, a well-recognised phenomenon where difficulties that are experienced by a child with a genomic disorder are interpreted as wholly due to ID [17–19]. This can reduce the chance for referral to appropriate services and access to appropriate treatment [20, 21].

One solution to these problems would be to identify patterns of neurodevelopmental and physical health symptoms that are most associated with ND-GCs, to develop a screening tool to stratify affected patients for graded approaches to investigation and treatment. Such a tool would need to be quick and simple to use either by a primary carer before consultations, or as part of a consultation, in a busy clinical setting, and focus on the most salient symptoms that could indicate future difficulties.

In the present study, we used a relatively large sample combining young people with a range of ND-GCs and siblings with no ND-GC (controls) in all of whom deep physical and mental health phenotyping had been conducted. We identify those symptoms that most robustly differentiate between young people with ND-GCs and controls and subsequently analysed whether these symptoms form broader symptom domains.

## Method

### Participants

We defined ND-GCs as conditions associated with increased risk of neurodevelopmental symptoms [22] and caused by a genetic variant which was either pathogenic or likely pathogenic, according to American College of Medical Genetics and Genomics guidance [23]. We aimed to recruit a population of participants with a range of ND-GCs that represented a “snapshot” of presentations to UK Child and Adolescent Mental Health Services, Intellectual Disability, Clinical Genetics or Community Paediatrics clinics.

Families of children with a confirmed ND-GC, aged over 4 years, were recruited through UK Medical Genetics clinics, word of mouth and the charities UNIQUE (https://rarechromo.org) and Max Appeal (https://www.maxappeal.org.uk), as part of ongoing cohort studies at Cardiff University including the ECHO study (https://www.cardiff.ac.uk/cy/centre-neuropsychiatric-genetics-genomics/research/themes/developmental-psychiatry/copy-number-variant-research-group) and the IMAGINE study (https://imagine-id.org) [22, 24]. Detailed information regarding the cohort inclusion criteria is available in the IMAGINE study protocol https://imagine-id.org/healthcare-professionals/study-documents-downloads-page/.

Siblings closest in age to individuals with a ND-GC, who did not have a known ND-GC themselves, were recruited to the study as controls; siblings were not excluded if they had any neurodevelopmental or physical health-related conditions.

In total, 589 individuals (441 individuals with a ND-GC and 148 siblings) were included in the study, from whom data from 493 individuals were included in our machine learning analysis after initial data preparation (Additional file 1: Methods). Participant demographic characteristics are shown in Table 1. Our sample size was the maximum number of participants in our dataset who had all the required variables.

**Table 1:** Demographic information about the sample of children affected by a ND-GC and sibling controls

Variable		Group	
	Overall, *N* = 493^a^	ND-GC, *N* = 389^a^	Sibling, *N* = 104^a^
Age	9.26 (7.27, 12.21)	9.01 (7.16, 11.82)	10.23 (8.12, 13.00)
Gender			
Female	182 (37%)	133 (34%)	49 (47%)
Male	311 (63%)	256 (66%)	55 (53%)
Highest educational level			
No school leaving exams	32 (6.5%)	29 (7.5%)	3 (2.9%)
Low	104 (21%)	86 (22%)	18 (17%)
Middle	175 (35%)	140 (36%)	35 (34%)
High	129 (26%)	105 (27%)	24 (23%)
Unknown	53 (11%)	29 (7.5%)	24 (23%)
Income			
≤ £19,999	123 (25%)	105 (27%)	18 (17%)
£20,000–£39,999	166 (34%)	134 (34%)	32 (31%)
£40,000–£59,999	74 (15%)	62 (16%)	12 (12%)
£60,000+	71 (14%)	52 (13%)	19 (18%)
Unknown	59 (12%)	36 (9.3%)	23 (22%)
Ethnicity			
European	439 (89%)	356 (92%)	83 (80%)
Other	31 (6.3%)	26 (6.7%)	5 (4.8%)
Unknown	23 (4.7%)	7 (1.8%)	16 (15%)

^a^Median (IQR); *n* (%)

Informed, written consent was obtained prior to recruitment from the carers of participants and recruitment was carried out in agreement with protocols approved by relevant NHS and university research ethics committees. Individual ND-GC genotypes were established from medical records and in-house genotyping at the Cardiff University Centre for Neuropsychiatric Genetics and Genomics using microarray analysis. The ND-GCs of participants are shown in Table 2.

**Table 2:** Counts of the genotypes of all study participants

Genomic condition	*N*
Controls	104
Other^a^	81
16p11.2 deletion	45
15q11.2 deletion	39
22q11.2 deletion	30
1q21.1 duplication	28
16p11.2 duplication	25
15q13.3 deletion	24
22q11.2 duplication	23
15q13.3 duplication	20
1q21.1 deletion	18
NRXN1	16
TAR duplication	13
16p11.2 distal deletion	11
Kleefstra	11
15q11.2 duplication	5

^a^To preserve the confidentiality of individuals who had ND-GCs with a total count of < 5 participants with the same ND-GC in the study, we have grouped all such low frequency ND-GCs into a single group. This group contained 32 deletions and 25 duplications, with 15 other conditions being related to mixed deletions and duplications, single nucleotide variants, triplications, translocation, chromosomal trisomy, or imprinting. Chromosomal regions affected by ND-GCs in this group were: 1p21, 1p33, 1p36, 1q21, 1q42, 1q44, 2p12, 2p16, 2q11-q21, 2q13, 2q33, 2q34, 2q37, 3q28-29, 4p15, 4q28-31, 5p15, 5q23, 6p25, 6q27, 7p22, 7q11, 8q21, 8q24, 9p24, 9q34, 11q23, 12p13, 15pter-q13, 15q11, 15q11-q13, 15q13, 16p11, 16p12, 16p13, 16p21, 16q23, 17p11, 17p13, 17q12, 17q23, 17q25, 18p11, 20q13, 22q11, 22q12-q13, 22q13, Xp21, Xp22, Xp28

### Assessments

Primary carers of participants completed a battery of assessments to collect comprehensive information on physical and mental health problems through semi-structured interviews with trained research staff and questionnaires. Assessments were carried out between January 2011 and December 2019.

Our goal was to generate a set of discriminating items that could be quickly, easily and conveniently completed by a carer or community clinician either on paper or online, and which could serve as the basis for the development of an instrument screening for the most likely domains in which young people with ND-GCs can experience difficulties. Therefore, measures which involved complex or prolonged assessments, such as IQ or motor co-ordination, or potentially intrusive testing, such as blood tests, although important for a full and in-depth assessment of phenotype in some settings, were not included in the current analysis.

Psychiatric symptoms were measured using the Child and Adolescent Psychiatric Assessment (CAPA, [25]), Strengths and Difficulties Questionnaire (SDQ, [26]) and the Social Communication Questionnaire (SCQ, [27]). The CAPA assesses a broad set of psychopathological domains including ADHD, anxiety disorders, oppositional defiant disorder, obsessive compulsive disorder, psychosis and psychotic experiences, tic disorders, mood disorders, and substance abuse. The SDQ is a dimensional measure of psychopathology that includes measures of hyperactivity, emotional problems, peer problems, and prosocial behaviour. The SCQ measures ASD-associated symptoms and was used as the CAPA and SDQ lack of coverage of ASD symptoms.

Difficulties with coordinated movement are also an important symptom in individuals with ND-GCs [10, 24, 28, 29]; therefore, we assessed motor coordination using the developmental coordination questionnaire (DCDQ, [30]).

Information about physical health problems and development was collected through a detailed questionnaire covering developmental history including pregnancy and birth and health problems in all major organ systems. A full list of all gathered variables is available on the IMAGINE ID study website https://imagine-id.org/wp-content/uploads/2019/04/Online-Data-dictionary-16.04.19-v2.pdf.

Included items were selected to cover a wide set of domains, including neurodevelopmental disorders, psychopathology more broadly, general health and development, motor development, social and communication skills and areas of strength and prosocial skills.

After variable filtering for excessive similar responses and missing data, all but one variable (birth weight in kg) was either binary or ordinal. We therefore did not perform any transformation on our variables.

### Statistical analysis and data availability

**Fig. 1:**
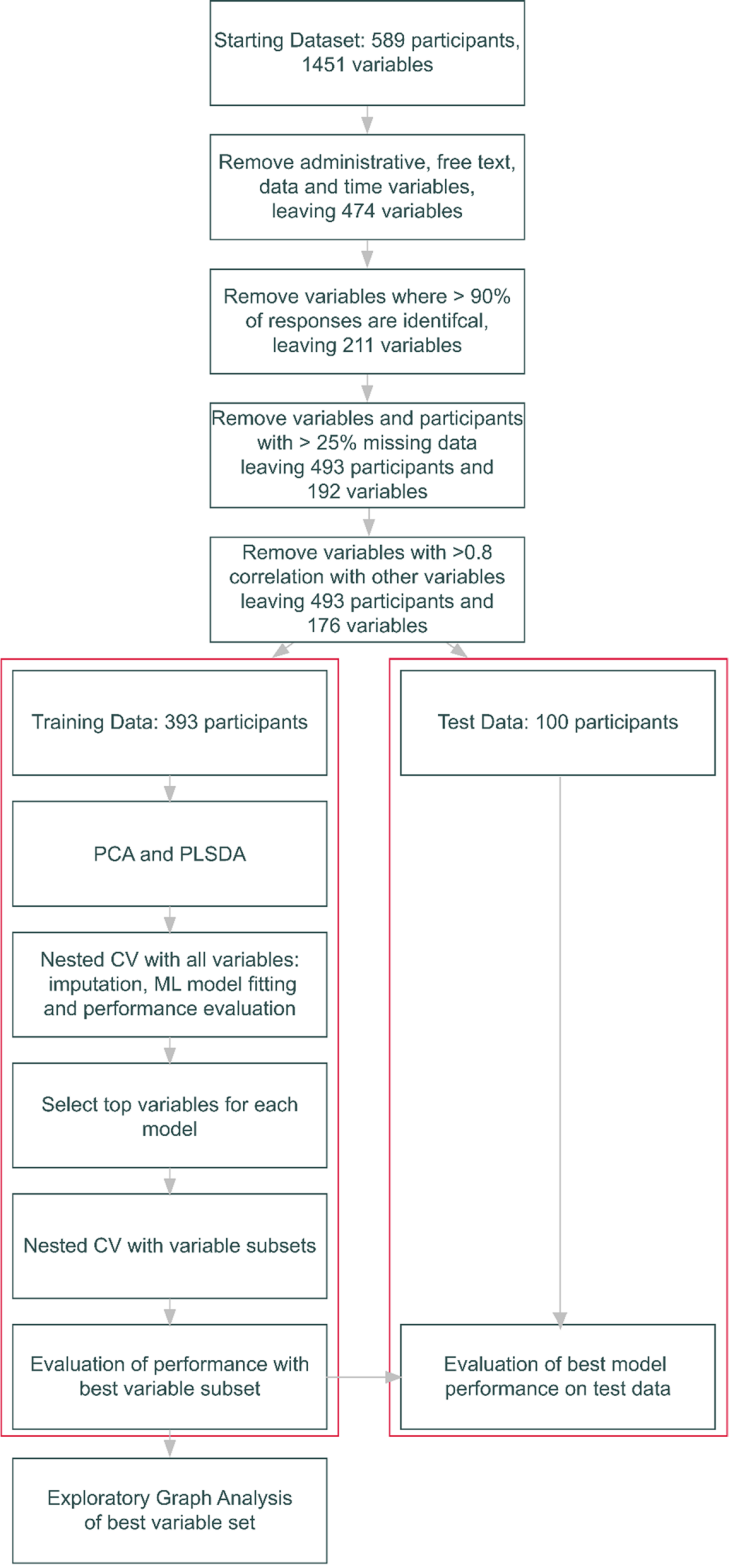
Flowchart of analysis workflow including variable and participant selection and machine learning model fitting. CV: cross-validation; ML: machine learning; PCA: principal components analysis; PLSDA: partial least squares discriminant analysis

All statistical analyses were carried out in R version 4.2.1 [31]. An overview of the analysis workflow is presented in Fig. 1. Code used in the project is provided in a GitHub repository: https://github.com/NADonnelly/nd_cnv_ml and fitted models are presented as an interactive Shiny app: https://nadonnelly.shinyapps.io/cnv_ml_app/. Data from the IMAGINE study are available via the IMAGINE ID study website: https://imagine-id.org/healthcare-professionals/datasharing/. Analysis is reported in line with the TRIPOD guidelines, Additional file 1: Table S1 [32]. An early version of this manuscript was deposited as a preprint: 10.1101/2022.12.16.22283581.

### Dimensional structure assessment

We applied principal components analysis (PCA) followed by partial least squares discriminant analysis (PLSDA, where the outcome was ND-GC status) to explore the dimensional structure of our dataset, using the *mixOmics* package [33]. A cross-validation process was used find the optimal number of components and variables for the PLSDA (Additional file 1: Methods).

### Machine learning model fitting

We prepared our data for machine learning (ML) model fitting by splitting participants into a training dataset of 393 (80% of the dataset) and a test set of 100 (20% of the dataset), stratifying by ND-GC status, sex and age (categorised into quintiles). The distribution of demographic characteristics in the test and training sets was reasonably balanced (Additional file 1: Table S2).

Our outcome was binary classification of ND-GC status (with ND-GC vs control), and we evaluated model performance using the area under the receiver operator characteristic curve (AUROC) and Brier Score (mean squared error between predicted probability and true ND-GC status, where controls were scored as 0 and individuals with an ND-GC as 1).

We used penalised logistic (elastic net) regression (using the *glmnet* package [34]), random forests (using the *Ranger* package [35]), radial basis function support vector machines (SVMs, using the *kernlab* package [36]) and single layer artificial neural networks (using the *nnet* package [37]) to create models capable of capturing linear and nonlinear relationships.

Models were fit using nested cross-validation (CV), with 20 outer folds and 20 inner folds. Outer folds were generated by splitting the data into 5 folds, repeated 4 times. Inner folds were generated from the outer fold analysis set using bootstrapping with replacement.

Within each outer fold missing data were imputed using bagged tree models [38], and the same model was used to impute missing data in the analysis set.

Grid search (30 elements) was used to optimise hyperparameters for ML models across inner folds. Model performance was evaluated by fitting the model with the best performing set of hyperparameters in the inner fold data to the (previously unseen) outer fold assessment dataset. This process was then repeated for all outer folds (Additional file 1: Methods).

As an additional analysis, as our dataset was imbalanced with regard to ND-GC status, we also trained and evaluated machine learning models after either downsampling the number of individuals with ND-GCs to be equal in number of controls; or upsampling control individuals to be equal in number to those with ND-GCs, using random resampling with replacement.

Following nested CV, we selected models with the highest AUROC, and evaluated the importance of all included variables for model prediction using permutation testing [39]. We selected the top 30 variables for all ML models and generated two further variable sets: all variables which were included in the top 30 most important for more than one ML model, and those variables included in the top 30 for at least 3 models, to give a total of 6 sets of variables.

We extracted 30 variables for each model because we wanted to achieve a balance between accurate prediction, including a wide set of variables for exploration of dimensional structure and limiting the number of items to that which could be realistically completed by young people’s carers and/or clinicians as a brief screening tool to be used in a clinical setting.

We repeated our nested CV process using the same ML models using the 6 sets of most-predictive variables, giving a total of 24 combinations of models and predictor variables, selecting the best performing combinations of variables and ML model, based on AUROC.

We evaluated the performance of the final models using the held-out training data. Missing data in the test dataset was imputed using a model fit to the full training dataset, and the ND-GC status of each participant in the test dataset was predicted using the best ML models.

Model performance was evaluated by drawing 2000 bootstrap samples from the test dataset and estimating performance (AUROC and Brier Score) for the bootstrap sample. This produced a distribution of values from which a median value and a 95% confidence interval were calculated.

Model calibration, i.e. the relationship between true and model-predicted probability of ND-GC status, was estimated by binning model predictions by predicted probability of ND-GC status and plotting this against true ND-GC status. Model performance was also estimated for male and female participants separately, and after binning participants by age quintile.

The importance of each variable in the best fitting model was evaluated using a permutation-based approach, as above.

The optimal threshold for converting model predicted probability of ND-GC status into a binary classification was estimated by finding the threshold which maximised the j-index (sensitivity + specificity – 1, [40]).

### Exploratory graph analysis

Bootstrap exploratory graph analysis (EGA) was used to investigate the dimensional structure of the best performing variable set. EGA has been shown to be as accurate or more accurate than traditional factor analytic methods such as parallel analysis [41]. Bootstrap EGA estimates and evaluates dimensional structure in a set of variables by first applying a network estimation method (*EBICglasso* as applied using the *qgraph* package [42]), followed by a community detection algorithm for weighted networks (Walktrap community detection algorithm [43]). Nonparametric bootstrapping is then used to generate bootstrap samples (*n* = 9999) from the input dataset, and EGA was applied to each replicate sample to form a sampling distribution from which the median value of each edge across the replicate networks, resulting in a single network. The stability of the network can be assessed by measuring the proportion of bootstrapped networks where a given variable is included in each putative dimension [44], and the number of variables included can be adjusted to improve the stability of dimension representations. We therefore fit an EGA model to a full set of variables, then repeated the analysis with the variables with the most consistent relationship to our dimensions (item stability > 0.75; this left 19 variables), generating a stable and consistent EGA model.

To provide an additional assessment of the fit of the proposed dimensional structure to the data, confirmatory factor analysis was carried out on the typical dimension structure identified by bootstrap EGA, with fit assessed using the comparative fit index (CFI) and root mean square error of approximation (RMSEA).

Finally, we repeated the above model fitting processing using the most important variables in each of the five dimensions identified by EGA.

## Results

### Study participant characteristics

A total of 493 participants contributed to our dataset, including 389 young people with a ND-GC and 104 controls. Demographic characteristics of study participants are given in Table 1 and genotypes in Table 2. Individuals with an ND-GC were approximately a year younger than controls and there was a higher proportion of males in the ND-GC group. Compared to families where both a control and a young person with a ND-GC took part, families where only a young person with a ND-GC took part had lower parental educational level and income, and there were fewer participants of European ancestry; the discrepancy between individuals with ND-GCs and control individuals was due to most young people with a ND-GC not having a sibling included in the study (58%).

### Partial least squares analysis

We applied principal components analysis (PCA) and partial least squares discriminant analysis (PLSDA) to our full set of 176 variables for the 389 participants in our training dataset to describe the dimensional structure of our variables. PLSDA is a supervised dimension reduction method which focusses on discrimination between groups. We found that 2 components provided optimal discrimination between groups, with 50 and 40 variables selected for the two components, respectively. This analysis indicated the two components explained 14.8% and 5.4% of the variance in our dataset (Additional file 1: Fig. S1). This analysis indicated that it was possible to identify young people with ND-GCs using our dataset; young people with ND-GCs had lower scores on component 1. Some individuals with a ND-GC showed similar profiles to controls and likely represent participants with a ND-GC that are relatively mildly affected; some controls showed profiles more like those with ND-GCs, reflecting individuals in the control sample with elevated difficulties across the measured domains.

However, this analysis still selected large numbers of variables. We therefore applied machine learning approaches to develop classification models that identified an optimally predictive subset of variables.

### Developing machine learning models

We developed machine learning models (artificial neural networks [ANN], radial basis function support vector machines [SVM], penalised logistic regression [LR] and random forests [RF]) to classify individuals by ND-GC status, using our full training set of 176 variables and 393 participants using nested cross-validation (CV). After nested CV, all models performed well at distinguishing between individuals in the training data set with a ND-GC and controls, with median AUROCs ≥ 0.9 in all cases (Additional file 1: Table S3). The RBF SVM performed best, with an overall median AUROC of 0.934 95% credible interval [0.914, 0.953]. The random forest and penalised logistic regression models did not perform significantly worse than the SVM, but the performance of the ANN was poorer (AUROC difference = − 0.02, 95% credible interval of difference [− 0.031, − 0.009]).

### Predictive performance with optimised variable sets

**Fig. 2:**
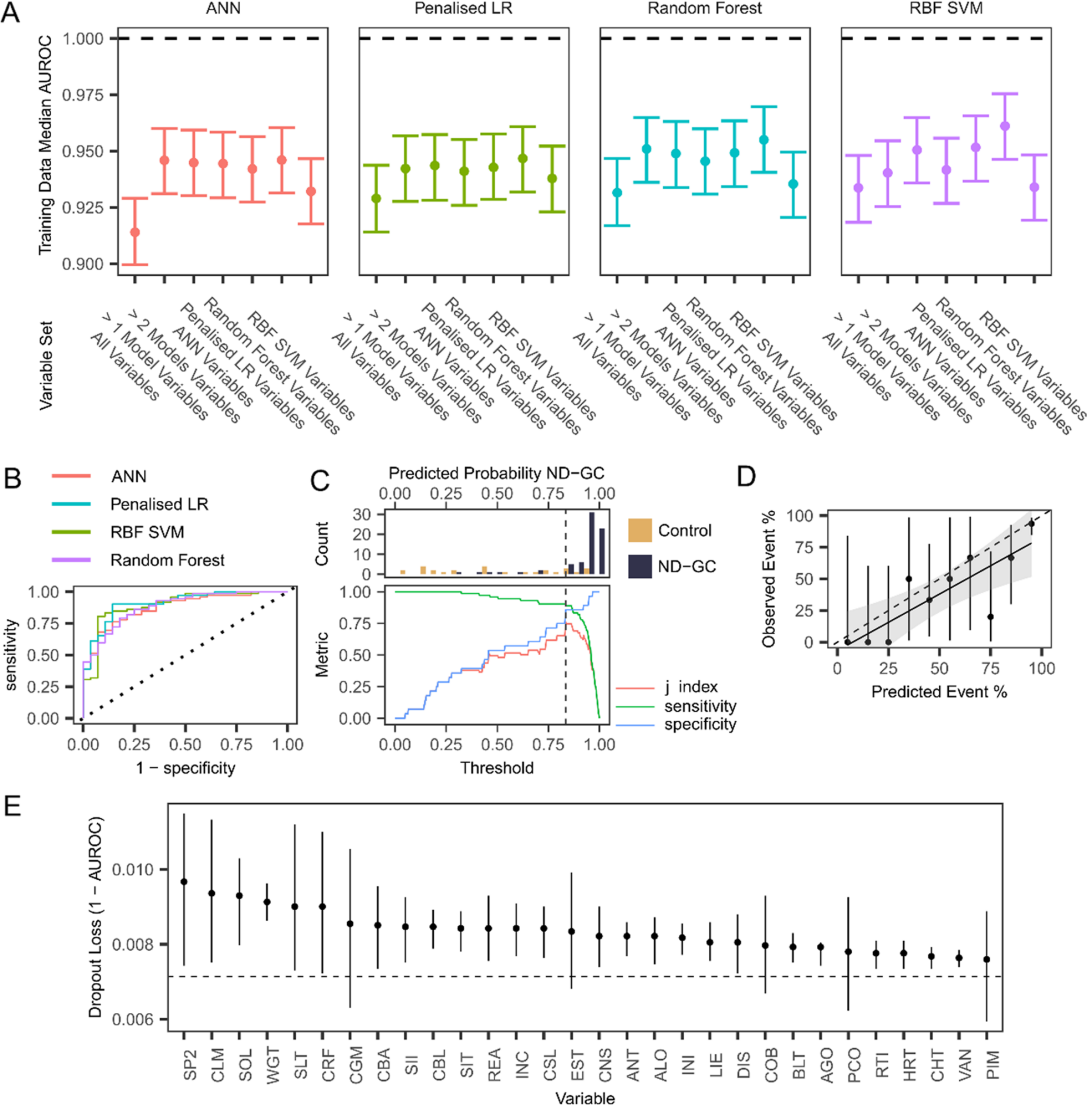
Performance of final models on test data. **A** Plot of performance (AUROC) of four ML models (ANN = artificial neural network, penalised LR = penalised logistic regression, random forest, RBF SVM = radial basis function support vector machine) fit to 7 variable sets (all variables = all 176 variables; ANN = 30 most important variables in an ANN fit to all variables; penalized LR = 30 most important variables in a penalized logistic regression fit to all variables; random forest = 30 most important variables in a random forest model fit to all variables; > 1 Model = variables identified as being in the 30 most important variables by more than one ML model; > 2 Models = variables identified as being in the 30 most important variables by more than two ML models; SVM = the 30 most important variables in a Radial Basis Function SVM fit to all variables. Points show the median posterior AUROC, error bars show the 95% credible interval of the AUROC. **B** Receiver-operator characteristic curves for the 4 machine learning models, using the 30 variables from the random forest dataset. **C** Top—histogram of predicted probability of ND-GC status in the 100 participants in our testing dataset using the best performing random forest model; bottom—plots of sensitivity, specificity of model classification performance at different thresholds for categorising a predicted probability. **D** Calibration plot for the best performing RF model. Points are performance in each decile, vertical lines show 95% confidence intervals, thick diagonal line shows a linear model fit to the data, with the shade area showing the 95% confidence interval of the linear model. A perfectly performing model would follow the diagonal dashed line. **E** Variable importance for the best fitting model. Mean dropout loss is the mean change in model AUROC after a given variable is permuted (repeated 500 times). Horizontal line indicates (1—AUROC) of the full model; therefore, variables with mean values above this line have a negative impact on model fit when permuted. Variable definitions are provided in Additional file 1: Table S7

We repeated model fitting using nested cross-validation using the sets of variables selected as being most important to the models fit to the full set of variables (determined using permutation testing). Results were similar across multiple models and variable sets (median training performance ranged from 0.914 to 0.961, Fig. 2A, Additional file 1: Table S4). We selected the “RF” variable set for further analysis as this set appeared to produce both the best classification performance across multiple model types.

We assessed whether model performance was altered by up- or down-sampling our training datasets such that the training data was balanced for status ND-GC. This analysis indicated that there were only minor changes in performance after up or downsampling (Additional file 1: Table S5). We therefore carried out all further analyses with the original training dataset.

We then fit the best performing models to our held-out test set of data from 100 participants (Fig. 2B, Table 3). The best performing model was a RF, achieving an AUROC of 0.915 (95% bootstrapped CI [0.838, 0.980]) with a Brier Score of 0.188 (95% bootstrapped CI [0.121, 0.243]).

**Table 3:** Final model performance on held-out test dataset

Model	Brier score	AUC ROC	AUC ROC difference	Probability of direction
Random forest	0.188 [0.121, 0.243]	0.915 [0.838, 0.98]	–	–
Penalised LR	0.183 [0.121, 0.251]	0.904 [0.82, 0.981]	0.011 [− 0.099, 0.122]	0.843
ANN	0.186 [0.152, 0.225]	0.883 [0.787, 0.963]	0.031 [− 0.087, 0.151]	0.619
RBF SVM	0.21 [0.137, 0.284]	0.897 [0.814, 0.968]	0.018 [− 0.089, 0.124]	0.757

Values shown are bootstrapped performance and the 95% confidence interval of the measure (AUROC and Brier Score), and difference in AUROC between the random forest and other ML models, with its 95% confidence interval, and the probability of direction for the AUROC difference

Performance of other models was not significantly poorer than the RF. The optimal probability for classifying a participant as having an ND-GC, the point at which the j-index is maximised, was 0.835 (Fig. 2C). Using this point as the cut off for classification, the RF model correctly classified 65/72 young people with ND-GCs (90.3%) and 24/28 controls (85.7%).

We investigated whether classification performance varied over participant age or between genders. Performance of the final RF model appeared to be higher in male than female participants, but there did not appear to be consistent differences in performance across participant ages, although our sample was mostly of younger participants (Additional file 1: Table S6).

Analysis of model calibration demonstrated miscalibration between predicted and actual probabilities, with the model having some tendency to given lower-than-optimal predicted probabilities of ND-GC status (Fig. 2D).

We investigated variable importance in our best performing model (Fig. 2E). This demonstrated that a subset of variables appeared to have a particularly large importance to the model. We next investigated whether there was a dimensional structure within our variable set that could be used to understand the predictors of ND-GC status.

### Underlying dimensional structure of selected variables

We next investigated an underlying structure of the variables included using an exploratory graph analysis (EGA). The 30 variables used were the optimised variable set of the best performing RF model, determined using permutation testing. These variables included items from the Developmental Coordination Disorder Questionnaire, Social Communication Questionnaire, Social Communication Questionnaire, Child and Adolescent Psychiatric Assessment and the Health and Development Questionnaire.

**Fig. 3:**
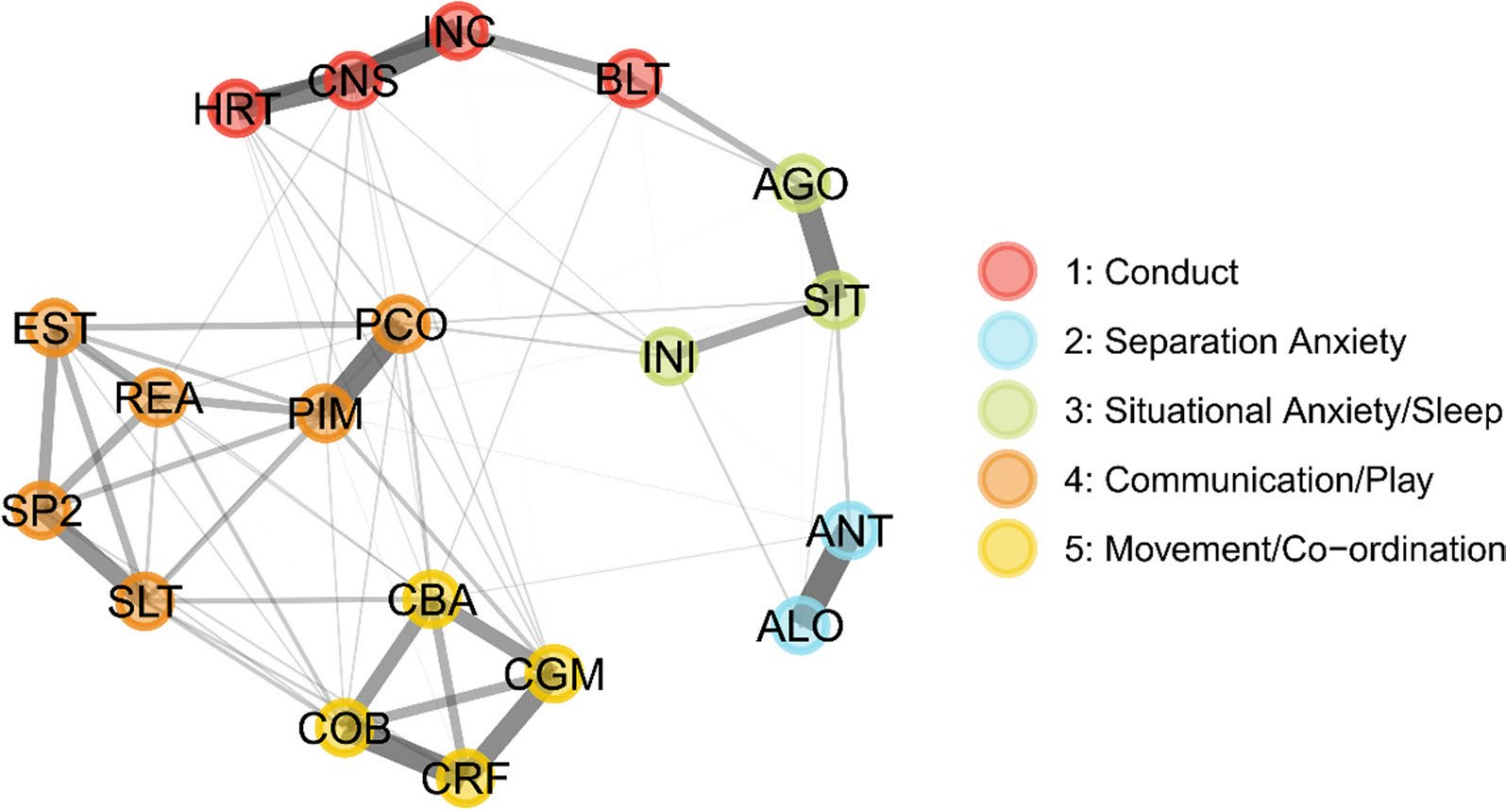
Exploratory graph analysis. The graph shows correlations between variables (notes) as lines, where line thickness represents correlation strength (range 0–1). Nodes are coloured by the putative dimensions they are assigned to by the bootstrapped EGA algorithm. Variable definitions are given in Additional file 1: Table S7.

EGA fit to the most stable set of variables (19 variables were included in the final EGA model) revealed that the variables formed a structure consisting of 5 dimensions: 1: conduct; 2: separation anxiety; 3: situational anxiety and insomnia, 4: communication; and 5: co-ordination (Fig. 3, Additional file 1: Table S7).

Confirmatory factor analysis based on this four-dimension structure demonstrated that the 4-factor structure fit with RMSEA of 0.046 and CFI of 0.980, indicating satisfactory fit to the data.

Finally, we investigated if the variable domains identified through EGA could be used to develop a further reduced set of variables for use in a ML model; although a 30-item scale could be realistically used in a clinical setting, a shorter screener could be useful in busy clinical environments. We therefore selected the variable in each dimension with the highest variable importance and fit ML models to our training data, using these variables (AGO [agoraphobia intensity], ANT [anticipatory distress intensity], BLT [blurting out answers to questions], SP2 [talking by age 2], CGM [participating in sports or games]).

The best performing model was a penalised logistic regression model with AUROC = 0.859 (bootstrapped 95% CI [0761, 0.955]) and Brier Score = 0.247 [0.203, 0.292]. Sensitivity and specificity were maximised at a threshold of 0.763; with 64/72 participants with an ND-GC being correctly classified (88.9%), and 19/28 control participants classified correctly (67.9%). This performance was lower than the full 30 variable model, but still indicative of reasonable classification performance.

## Discussion

### Main findings

In this study, we demonstrate the potential of using machine learning to identify key variables where individuals with genomic conditions associated with intellectual disability and neurodevelopmental disorders differ from control individuals, based on a limited set of psychiatric, behavioural and physical health related variables, in the absence of biochemical, genetic or neurocognitive data. Using a random forest classifier, we were able to classify individuals with an ND-GC with excellent performance, achieving an AUROC of 0.915. We identified 5 dimensions in our variable set that appeared to be most relevant to identifying individuals with an ND-GC, namely conduct, separation and situational anxiety, communication and motor co-ordination.

### Relationship to previous studies

Previous studies have described the high rates, and complex presentations, of psychiatric and neurodevelopmental difficulties in young people with ND-GCs [8, 12, 22, 24, 45]. ND-GCs are associated with a wide range of health outcomes [15], along with multi-morbidity later in life [46], and are highly enriched in the population with developmental delay/intellectual disability [1, 3, 4, 47]. However, not all individuals with a ND-GC will meet diagnostic criteria for specific psychiatric disorders [48]. We attempted to address this by not including diagnostic status in our classification models, only symptom scores; the highly accurate classification we were able to achieve supports the idea that profiles of symptoms are most informative when identifying areas of relative difficulty or strength in individuals with ND-GCs.

We identified 5 underlying dimensions in our final set of variables. These dimensions identify potential key phenotypic areas where individuals with ND-GCs differ from controls: anxiety (particularly separation anxiety) and insomnia, motor co-ordination, communication skills and conduct, as well as suggesting that other domains, such as difficulties with hyperactivity, may be less discriminating. The identified dimensions map onto areas of difficulty elucidated in previous studies [11, 28, 48–51], and highlight that specific symptoms may be particularly informative about ND-GC status, including symptoms of separation anxiety and difficulties with speech.

Clinical care pathways may be enhanced by focusing more on the areas identified as key dimensions by our analysis if further research demonstrates that they are areas that predict longer term difficulties for children with ND-GCs. It will also be important to take the items identified and work with parents and clinicians to optimise the wording and content of any items that could be used in a screening test derived from our analysis. For example, two highly predictive items refer to a history of speech and language therapy or having an education statement of needs from a school. As young people with ND-GCs can struggle to access therapies in a timely fashion, this item might miss individuals who might have needed speech and language therapy, but not been able to access it; similarly, there may be delays to accessing support in schools; therefore, asking about relative difficulties with speech and language may be more informative.

### Strength and limitations

This is the largest study of its kind to investigate the possibility of identifying domains of differences in presentation in individuals with a broad range of ND-GCs based solely on psychiatric and health phenotypes using machine learning models. We were able to produce a model with high AUROC, which performed well across a range of relevant ages, and in both males and females.

However, while including a very broad range of genomic disorders provided a more representative sample of those variants which may be seen by clinical services, it may have increased the noise and variability in symptom profiles. This requires empirical testing.

Similarly, we included siblings as controls based on genetic testing confirming the absence of an ND-GC, rather than based on phenotype. Our sample was also unbalanced, in that there were a larger number of individuals with a ND-GC than controls, because not all families with a child with an ND-GC had an sibling of a similar age at recruitment, and our dataset is derived from a cohort study that specifically aims to recruit individuals with ND-GCs. This can affect model performance, as most techniques perform best in balanced samples. Although we performed additional sensitivity analysis demonstrating that model performance remained similar when either upsampling controls or downsampling individuals with ND-GCs, future studies that include larger sets of controls, in both siblings and unrelated typically developing individuals, will be important for validating our models.

Our initial partial least squares discriminant analysis indicated that young people with an ND-GC and control individuals lie on a spectrum of presentations; while it is possible to distinguish between the two groups based on psychiatric, behavioural and health information, there remain some individuals with a ND-GC who have profiles that are very similar to control individuals. This highlights the wide variety of phenotypic expression that is seen within individuals with ND-GCs, which will impose limits on the performance of any classification algorithm.

Additionally, ascertainment bias may affect our results. Developmental delay is a major reason for referral for genetic testing in the UK, and it is likely that our sample has a preponderance to include those individuals with ND-GCs who are on the more severe end of the phenotypic presentation, and as such it may be the case that the dimensional structure we identify as being associated with ND-GC carriage may be applicable only to relatively more severe difficulties, rather than the phenotype of the entire population of young people with ND-GCs.

We considered the role of decision curve analysis in our study, as this approach has been recommended in studies of prediction models [52]. However, such calculations rely on samples being drawn from a population comparable to the clinical population. Our study sample was drawn from a cohort explicitly recruited based on a positive test for a ND-GC (or sibling controls). Therefore, such an analysis is not applicable to our study. However, it should be performed in a future study validating our model in a broader population.

Our machine learning models and EGA would be strengthened by measuring performance and performing confirmatory factor analysis using an independent sample. Future studies which combine measurement of most differentiating variables and longer-term follow-up of psychiatric and health outcomes would allow the predictive accuracy of our model to be evaluated.

We included only items that were reported by participant’s parents or carers, rather than from participant self-report, or from other sources of information such as teacher report or clinical observation. Although multi-informant and multi-modal assessment would be the gold standard for accurate diagnosis, parental report is more likely to be available in many clinics as a starting point to identify individuals who require more detailed assessment.

The symptom domains identified could be explored in future work by, as suggested, the development of self-reporting tasks, or the use of novel technology such as analysis of video recordings using machine learning algorithms (for example given our finding that communication and motor co-ordination are important domains) or ecological momentary assessment methods.

Despite these limitations, it is important to better understand the difficulties faced by this group of individuals as they make up a significant proportion of those presenting to intellectual disability services and clinicians often lack complete information on prognosis for patients with ND-GCs. This study highlights areas of difficulties for those children who may most need further support, which may warrant further research and may be targets for individualised interventions.

## Conclusions

We develop a set of questionnaire variables associated with neurodevelopmental disorders and intellectual disability symptoms in ND-GCs which could form the basis for clinical screening instruments. We highlight that conduct, separation and situational anxiety, communication and motor skills and conduct are important areas where children with ND-GCs differ from control individuals. Future research should investigate the prognostic associations of difficulties in these domains.

## Supplementary Information

**Additional file 1** contains supplementary methods, supplementary tables and a supplementary figure.

## Data Availability

Code used in the project is provided in a GitHub repository: https://github.com/NADonnelly/nd_cnv_ml, and fitted models are presented as an interactive Shiny app: https://nadonnelly.shinyapps.io/cnv_ml_app/. Data from the IMAGINE study are available via the IMAGINE ID study website: https://imagine-id.org/healthcare-professionals/datasharing/.

## Abbreviations

ADHD: Attention deficit hyperactivity disorder
ANN: Artificial neural networks
ASD: Autism spectrum disorder
AUROC: Area under the receiver operator characteristic curve
CAPA: Child and Adolescent Psychiatric Assessment
CFI: Comparative fit index
CV: Cross-validation
DCDQ: Developmental coordination questionnaire
EGA: Exploratory graph analysis
ID: Intellectual disability
LR: Penalised logistic regression
ML: Machine learning
ND-GC: Neurodevelopmental genomic conditions
PCA: Principal components analysis
PLSDA: Partial least squares discriminant analysis
RF: Random forest
RMSEA: Root mean square error of approximation
SCQ: Social Communication Questionnaire
SDQ: Strengths and Difficulties Questionnaire
SVM: (Radial basis function) Support vector machine
