# Supplementary material for: Identifying the neurodevelopmental and psychiatric signatures of genomic disorders associated with intellectual disability: a machine learning approach
Source: Mol Autism. 2023 May 23;14:19. doi: 10.1186/s13229-023-00549-2 (PMC10207854; doi:10.1186/s13229-023-00549-2)
Supplement: Supplementary file 1 — Additional file 1 contains supplementary methods, supplementary tables and a supplementary figure. [file 13229_2023_549_MOESM1_ESM.docx]

# Additional file 1

# Title Page

## Article Title

Identifying the neurodevelopmental and psychiatric signatures of genomic disorders associated with intellectual disability: a machine learning approach

## Authors

Nicholas Donnelly^1,2^ (ORCID: 0000-0003-2234-8545),

Adam Cunningham^3^ (ORCID: 0000-0002-9791-7813),

Sergio Marco Salas^3^ (ORCID: 0000-0002-4636-0322),

Matthew Bracher-Smith^3^ (ORCID: 0000-0002-0531-6309),

Samuel Chawner^3^ (ORCID: 0000-0002-2590-2874),

Jan Stochl^4,5^ (ORCID: 0000-0002-9693-9930),

Tamsin Ford^4^ (ORCID: 0000-0001-5295-4904),

F Lucy Raymond^6^ (ORCID: 0000-0003-2652-3355),

Valentina Escott-Price^3^ (ORCID: 0000-0003-1784-5483),

Marianne BM van den Bree^3^ (ORCID: 0000-0002-4426-3254)

*^1^Centre for Academic Mental Health, Population Health Sciences, University of Bristol, UK*

*^2^ MRC Integrative Epidemiology Unit, Population Health Sciences, Bristol Medical School, University of Bristol, UK.*

*^3^ Centre for Neuropsychiatric Genetics and Genomics, Division of Psychological Medicine and Clinical Neurosciences, Cardiff University School of Medicine, Cardiff, UK.*

*^4^ Department of Psychiatry, University of Cambridge, Cambridge, UK*

*^5^ Department of Kinanthropology, Charles University, Prague, Czechia*

*^6^ Department of Medical Genetics, University of Cambridge, Cambridge, UK*

# Supplementary Methods

## Initial Variable Filtering

The initial dataset contained 1451 variables with information from 589 individuals (441 individuals with a ND-GC and 148 control individuals without an ND-GC). To prepare the data for analysis, we began by removing those variables that contained administrative, free text and date and time information, as well as variables that were not quantitative questionnaire responses or coding of symptom intensity. This left 474 variables.

Following these initial steps, variables where the most common response made up greater than 90% of responses to the question were removed as these items would likely not be useful in distinguishing young people with ND-GCs and mental health or behavioural difficulties from other young people. This left 211 variables.

Next, those variables with a missing data rate greater than 25% were removed, leaving 192 variables. Once the variables had been filtered, individuals with missing data rates across the remaining variables greater than 25% were also removed.

As a final variable filtering step, we removed highly correlated variables to maximise the independence of the remaining variable set. We calculated the polychoric correlation between all pairs of variables and removed variables with >0.8 correlation with others. Within a pair of correlated variables, the variable with the highest mean absolute correlation with all other variables was removed. These steps resulted in 493 individuals (389 ND-GC carriers [78.9%], 104 controls [21.1%]) and 176 variables retained for further analysis.

## Principal Components Analysis and Partial Least Squares Discriminant Analysis

To develop an initial understanding of the dimensional structure of our data, we applied principal components analysis (PCA) followed by partial least squares discriminant analysis (PLSDA) to our training dataset, using the R *mixOmics* package (1). We used PCA as an initial unsupervised approach to identify the number of components that explained variance in our measured variables. Next, we applied a supervised approach (where the outcome was ND-GC status): sparse PLSDA. The number of components retained and number of variables per component were selected using 5-fold cross-validation, repeated 50 times, finding the combination that minimised prediction distance using one-sided t-tests testing for significant differences in the mean error rate when components are added to the model. The final PLSDA model was then fit with the optimal number of components and variables.

## Model Evaluation

Penalised logistic (elastic net) regression models optimised penalty and mixture parameters; random forests used 1000 trees and optimised minimal node size and number of variables split at each node; Radial Basis Function SVM models optimised sigma, cost and margin parameters, neural network models optimised the number of hidden units, epochs and model penalty.

Model performance was evaluated for each outer fold by fitting the model with the best performing set of hyperparameters in the inner fold data to the (previously unseen) outer fold assessment dataset. This process was then repeated for all outer folds.

Following nested cross validation, we compared model performance based on the AUROC values for each outer fold, using a Bayesian linear mixed model fit with the R *rstanarm* package (2), where the outer fold identity was included as a varying intercept. From this model we calculated the performance of each model using the median of the posterior distribution, and the 95% credible interval using the highest density interval method. Models were then compared using the probability of direction method (3).

Variable importance in each model was determined using permutation testing. This approach randomly permutes data from each variable in turn and evaluates the change in model performance (i.e., change in AUROC) following permutation. This was repeated 500 times to give a distribution of changes in performance after permutation. Variables with greater importance to the model will cause larger drops in AUROC than variables with lower importance

# Supplementary Tables

## Supplementary Table 1

| **Section/Topic** | **Itm** | **Checklist Item** | **Page** |
| --- | --- | --- | --- |
| **Title and abstract** | | | |
| Title | 1 | Identify the study as developing and/or validating a multivariable prediction model, the target population, and the outcome to be predicted. | Title |
| Abstract | 2 | Provide a summary of objectives, study design, setting, participants, sample size, predictors, outcome, statistical analysis, results, and conclusions. | Abstract |
| **Introduction** | | | |
| Background and objectives | 3a | Explain the medical context (including whether diagnostic or prognostic) and rationale for developing or validating the multivariable prediction model, including references to existing models. | Introduction |
|  | 3b | Specify the objectives, including whether the study describes the development or validation of the model or both. | Introduction |
| **Methods** | | | |
| Source of data | 4a | Describe the study design or source of data (e.g., randomized trial, cohort, or registry data), separately for the development and validation data sets, if applicable. | Methods and Materials - Participants |
|  | 4b | Specify the key study dates, including start of accrual; end of accrual; and, if applicable, end of follow-up. | Methods and Materials - Assessments |
| Participants | 5a | Specify key elements of the study setting (e.g., primary care, secondary care, general population) including number and location of centres. | Methods and Materials - Participants |
|  | 5b | Describe eligibility criteria for participants. | Methods and Materials - Participants |
|  | 5c | Give details of treatments received, if relevant. | N/A – observational study |
| Outcome | 6a | Clearly define the outcome that is predicted by the prediction model, including how and when assessed. | Methods and Materials - Participants |
|  | 6b | Report any actions to blind assessment of the outcome to be predicted. | N/A observational study |
| Predictors | 7a | Clearly define all predictors used in developing or validating the multivariable prediction model, including how and when they were measured. | Methods and Materials - Assessments |
|  | 7b | Report any actions to blind assessment of predictors for the outcome and other predictors. | N/A observational study |
| Sample size | 8 | Explain how the study size was arrived at. | Methods and Materials - Participants |
| Missing data | 9 | Describe how missing data were handled (e.g., complete-case analysis, single imputation, multiple imputation) with details of any imputation method. | Methods and Materials – Initial Variable Filtering and Machine Learning Model Fitting |
| Statistical analysis methods | 10a | Describe how predictors were handled in the analyses. | Methods and Materials – Initial Variable Filtering and Machine Learning Model Fitting |
|  | 10b | Specify type of model, all model-building procedures (including any predictor selection), and method for internal validation. | Methods and Materials – Machine Learning Model Fitting |
|  | 10d | Specify all measures used to assess model performance and, if relevant, to compare multiple models. | Methods and Materials – Machine Learning Model Fitting – paragraph 1 |
| Risk groups | 11 | Provide details on how risk groups were created, if done. | N/A |
| **Results** | | | |
| Participants | 13a | Describe the flow of participants through the study, including the number of participants with and without the outcome and, if applicable, a summary of the follow-up time. A diagram may be helpful. | Figure 1 |
|  | 13b | Describe the characteristics of the participants (basic demographics, clinical features, available predictors), including the number of participants with missing data for predictors and outcome. | Table 1 |
| Model development | 14a | Specify the number of participants and outcome events in each analysis. | Table 1 |
|  | 14b | If done, report the unadjusted association between each candidate predictor and outcome. | N/A |
| Model specification | 15a | Present the full prediction model to allow predictions for individuals (i.e., all regression coefficients, and model intercept or baseline survival at a given time point). | Results – Predictive performance with optimised variable sets; Shiny app <https://nadonnelly.shinyapps.io/cnv_ml_app/> |
|  | 15b | Explain how to the use the prediction model. | Results– Predictive performance with optimised variable sets; Shiny App <https://nadonnelly.shinyapps.io/cnv_ml_app/> |
| Model performance | 16 | Report performance measures (with CIs) for the prediction model. | Results – Developing machine learning models, Results – Predictive performance with optimised variable sets |
| **Discussion** | | | |
| Limitations | 18 | Discuss any limitations of the study (such as nonrepresentative sample, few events per predictor, missing data). | Discussion – Strengths and limitations |
| Interpretation | 19b | Give an overall interpretation of the results, considering objectives, limitations, and results from similar studies, and other relevant evidence. | Discussion – Main Findings, Discussion – Relationship to previous studies |
| Implications | 20 | Discuss the potential clinical use of the model and implications for future research. | Discussion - Conclusions |
| **Other information** | | | |
| Supplementary information | 21 | Provide information about the availability of supplementary resources, such as study protocol, Web calculator, and data sets. | Methods and Materials – Statistical Analysis |
| Funding | 22 | Give the source of funding and the role of the funders for the present study. | Funding statement |

**Supplementary Table 2 Caption**: *TRIPOD Reporting Guideline Table*

## Supplementary Table 2

| **Variable** |  | **Dataset** | |
| --- | --- | --- | --- |
|  | **Overall**, N = 493*^1^* | **Train**, N = 393*^1^* | **Test**, N = 100*^1^* |
| **Group** | | | |
| ND-GC | 389 (79%) | 317 (81%) | 72 (72%) |
| Control | 104 (21%) | 76 (19%) | 28 (28%) |
| **Age** | 9.26 (7.27, 12.21) | 9.33 (7.29, 12.20) | 9.18 (7.16, 12.51) |
| **Gender** | | | |
| Female | 182 (37%) | 145 (37%) | 37 (37%) |
| Male | 311 (63%) | 248 (63%) | 63 (63%) |
| **Highest** |  |  |  |
| No School Leaving Exams | 32 (6.5%) | 24 (6.1%) | 8 (8.0%) |
| Low | 104 (21%) | 80 (20%) | 24 (24%) |
| Middle | 175 (35%) | 145 (37%) | 30 (30%) |
| High | 129 (26%) | 104 (26%) | 25 (25%) |
| Unknown | 53 (11%) | 40 (10%) | 13 (13%) |
| **Income** |  |  |  |
| <=£19,999 | 123 (25%) | 92 (23%) | 31 (31%) |
| £20,000 - £39,999 | 166 (34%) | 133 (34%) | 33 (33%) |
| £40,000 - £59,999 | 74 (15%) | 64 (16%) | 10 (10%) |
| £60,000 + | 71 (14%) | 59 (15%) | 12 (12%) |
| Unknown | 59 (12%) | 45 (11%) | 14 (14%) |
| **Ethnicity** |  |  |  |
| European | 439 (89%) | 346 (88%) | 93 (93%) |
| Other | 31 (6.3%) | 29 (7.4%) | 2 (2.0%) |
| Unknown | 23 (4.7%) | 18 (4.6%) | 5 (5.0%) |
| *^1^* n (%); Median (IQR) | | | |

## Supplementary Table 3

| **Model** | **Performance** | **Difference** | **Probability of Direction** |
| --- | --- | --- | --- |
| RBF SVM | 0.934 [0.914, 0.953] | - | 1 |
| Random Forest | 0.931 [0.913, 0.951] | -0.002 [-0.013, 0.009] | 0.688 |
| Penalised LR | 0.929 [0.91, 0.948] | -0.005 [-0.015, 0.006] | 0.386 |
| ANN | 0.914 [0.895, 0.933] | -0.02 [-0.031, -0.009] | < 0.001 |

**Supplementary Table 3 Caption**: *Classification performance (AUROC) for each of the different machine learning techniques using training data and all variables. ANN: Artificial Neural Network; LR: Logistic Regression; SVM: Radial Basis Function Support Vector Machine*

## Supplementary Table 4

| Model | Variable Set | Performance | Difference | Probability of Direction |
| --- | --- | --- | --- | --- |
| Random Forest | Random Forest Variables | 0.955 [ 0.941 , 0.97 ] | - | - |
| Random Forest | All Variables | 0.932 [ 0.917 , 0.947 ] | -0.025 [ -0.047 , -0.004 ] | 0.024 |
| Random Forest | > 1 Model Variables | 0.951 [ 0.936 , 0.965 ] | -0.004 [ -0.011 , 0.004 ] | 0.294 |
| Random Forest | > 2 Models Variables | 0.949 [ 0.934 , 0.963 ] | -0.006 [ -0.014 , 0.001 ] | 0.11 |
| Random Forest | ANN Variables | 0.945 [ 0.931 , 0.96 ] | -0.009 [ -0.017 , -0.002 ] | 0.012 |
| Random Forest | Penalised LR Variables | 0.949 [ 0.934 , 0.963 ] | -0.006 [ -0.013 , 0.002 ] | 0.132 |
| Random Forest | RBF SVM Variables | 0.935 [ 0.921 , 0.95 ] | -0.02 [ -0.027 , -0.012 ] | 0 |
|  |  |  |  |  |
| ANN | Random Forest Variables | 0.946 [ 0.931 , 0.96 ] | -0.009 [ -0.017 , -0.002 ] | 0.016 |
| ANN | All Variables | 0.914 [ 0.9 , 0.929 ] | -0.042 [ -0.064 , -0.021 ] | 0 |
| ANN | > 1 Model Variables | 0.946 [ 0.931 , 0.96 ] | -0.009 [ -0.017 , -0.002 ] | 0.018 |
| ANN | > 2 Models Variables | 0.945 [ 0.93 , 0.959 ] | -0.01 [ -0.018 , -0.003 ] | 0.008 |
| ANN | ANN Variables | 0.944 [ 0.929 , 0.958 ] | -0.01 [ -0.018 , -0.003 ] | 0.006 |
| ANN | Penalised LR Variables | 0.942 [ 0.927 , 0.956 ] | -0.013 [ -0.02 , -0.005 ] | 0 |
| ANN | RBF SVM Variables | 0.932 [ 0.918 , 0.947 ] | -0.023 [ -0.03 , -0.015 ] | 0 |
|  |  |  |  |  |
| Penalised LR | Random Forest Variables | 0.947 [ 0.932 , 0.961 ] | -0.008 [ -0.016 , -0.001 ] | 0.028 |
| Penalised LR | All Variables | 0.929 [ 0.914 , 0.944 ] | -0.027 [ -0.048 , -0.006 ] | 0.014 |
| Penalised LR | > 1 Model Variables | 0.942 [ 0.928 , 0.957 ] | -0.013 [ -0.02 , -0.005 ] | 0 |
| Penalised LR | > 2 Models Variables | 0.944 [ 0.928 , 0.957 ] | -0.011 [ -0.019 , -0.004 ] | 0.004 |
| Penalised LR | ANN Variables | 0.941 [ 0.926 , 0.955 ] | -0.014 [ -0.021 , -0.006 ] | 0 |
| Penalised LR | Penalised LR Variables | 0.943 [ 0.929 , 0.958 ] | -0.012 [ -0.02 , -0.005 ] | 0.002 |
| Penalised LR | RBF SVM Variables | 0.938 [ 0.923 , 0.952 ] | -0.017 [ -0.025 , -0.01 ] | 0 |
|  |  |  |  |  |
| RBF SVM | Random Forest Variables | 0.961 [ 0.946 , 0.975 ] | 0.006 [ -0.001 , 0.014 ] | 0.114 |
| RBF SVM | All Variables | 0.934 [ 0.918 , 0.948 ] | -0.022 [ -0.043 , -0.001 ] | 0.038 |
| RBF SVM | > 1 Model Variables | 0.94 [ 0.925 , 0.955 ] | -0.015 [ -0.022 , -0.007 ] | 0 |
| RBF SVM | > 2 Models Variables | 0.95 [ 0.936 , 0.965 ] | -0.004 [ -0.012 , 0.003 ] | 0.24 |
| RBF SVM | ANN Variables | 0.942 [ 0.927 , 0.956 ] | -0.013 [ -0.021 , -0.006 ] | 0 |
| RBF SVM | Penalised LR Variables | 0.952 [ 0.937 , 0.966 ] | -0.003 [ -0.011 , 0.004 ] | 0.37 |
| RBF SVM | RBF SVM Variables | 0.934 [ 0.919 , 0.948 ] | -0.021 [ -0.029 , -0.014 ] | 0 |

**Supplementary Table 4 Caption**: *Classification performance for each of the different machine learning techniques using training data and different sets of variables. Column Performance is the median model performance over 20 outer folds of nested cross validation, estimated using a Bayesian generalised linear model, with 95% credible interval; Column Difference shows the model estimated difference in performance between a Random Forest model fit with the top 30 model important variables estimated by a Random Forest fit to all variables) and a given model.*

## Supplementary Table 5

| Model | Variable Set | Sampling | Performance |
| --- | --- | --- | --- |
| ANN | All Variables | Down | 0.917 [ 0.904 , 0.93 ] |
| ANN | All Variables | Simple | 0.913 [ 0.9 , 0.926 ] |
| ANN | All Variables | Up | 0.912 [ 0.899 , 0.924 ] |
| ANN | Penalised LR Variables | Down | 0.934 [ 0.921 , 0.947 ] |
| ANN | Penalised LR Variables | Simple | 0.941 [ 0.928 , 0.954 ] |
| ANN | Penalised LR Variables | Up | 0.933 [ 0.92 , 0.946 ] |
| ANN | > 2 Models Variables | Down | 0.939 [ 0.927 , 0.952 ] |
| ANN | > 2 Models Variables | Simple | 0.944 [ 0.932 , 0.957 ] |
| ANN | > 2 Models Variables | Up | 0.934 [ 0.921 , 0.947 ] |
| ANN | > 1 Model Variables | Down | 0.937 [ 0.924 , 0.95 ] |
| ANN | > 1 Model Variables | Simple | 0.945 [ 0.932 , 0.958 ] |
| ANN | > 1 Model Variables | Up | 0.939 [ 0.926 , 0.952 ] |
| ANN | ANN Variables | Down | 0.942 [ 0.929 , 0.955 ] |
| ANN | ANN Variables | Simple | 0.944 [ 0.931 , 0.957 ] |
| ANN | ANN Variables | Up | 0.938 [ 0.926 , 0.951 ] |
| ANN | Random Forest Variables | Down | 0.942 [ 0.93 , 0.955 ] |
| ANN | Random Forest Variables | Simple | 0.945 [ 0.932 , 0.958 ] |
| ANN | Random Forest Variables | Up | 0.947 [ 0.934 , 0.96 ] |
| ANN | RBF SVM Variables | Down | 0.928 [ 0.916 , 0.941 ] |
| ANN | RBF SVM Variables | Simple | 0.932 [ 0.919 , 0.944 ] |
| ANN | RBF SVM Variables | Up | 0.916 [ 0.904 , 0.929 ] |
|  |  |  |  |
| Penalised LR | All Variables | Down | 0.928 [ 0.915 , 0.941 ] |
| Penalised LR | All Variables | Simple | 0.928 [ 0.915 , 0.94 ] |
| Penalised LR | All Variables | Up | 0.934 [ 0.921 , 0.947 ] |
| Penalised LR | Penalised LR Variables | Down | 0.94 [ 0.928 , 0.953 ] |
| Penalised LR | Penalised LR Variables | Simple | 0.942 [ 0.929 , 0.955 ] |
| Penalised LR | Penalised LR Variables | Up | 0.942 [ 0.929 , 0.954 ] |
| Penalised LR | > 2 Models Variables | Down | 0.941 [ 0.928 , 0.954 ] |
| Penalised LR | > 2 Models Variables | Simple | 0.943 [ 0.931 , 0.956 ] |
| Penalised LR | > 2 Models Variables | Up | 0.945 [ 0.933 , 0.958 ] |
| Penalised LR | > 1 Model Variables | Down | 0.938 [ 0.924 , 0.95 ] |
| Penalised LR | > 1 Model Variables | Simple | 0.942 [ 0.929 , 0.955 ] |
| Penalised LR | > 1 Model Variables | Up | 0.939 [ 0.926 , 0.952 ] |
| Penalised LR | ANN Variables | Down | 0.941 [ 0.928 , 0.953 ] |
| Penalised LR | ANN Variables | Simple | 0.941 [ 0.928 , 0.953 ] |
| Penalised LR | ANN Variables | Up | 0.939 [ 0.926 , 0.952 ] |
| Penalised LR | Random Forest Variables | Down | 0.944 [ 0.931 , 0.956 ] |
| Penalised LR | Random Forest Variables | Simple | 0.946 [ 0.933 , 0.959 ] |
| Penalised LR | Random Forest Variables | Up | 0.946 [ 0.934 , 0.959 ] |
| Penalised LR | RBF SVM Variables | Down | 0.937 [ 0.924 , 0.95 ] |
| Penalised LR | RBF SVM Variables | Simple | 0.937 [ 0.924 , 0.95 ] |
| Penalised LR | RBF SVM Variables | Up | 0.939 [ 0.926 , 0.952 ] |
|  |  |  |  |
| Random Forest | All Variables | Down | 0.933 [ 0.92 , 0.946 ] |
| Random Forest | All Variables | Simple | 0.93 [ 0.918 , 0.943 ] |
| Random Forest | All Variables | Up | 0.937 [ 0.924 , 0.95 ] |
| Random Forest | Penalised LR Variables | Down | 0.949 [ 0.936 , 0.962 ] |
| Random Forest | Penalised LR Variables | Simple | 0.949 [ 0.936 , 0.961 ] |
| Random Forest | Penalised LR Variables | Up | 0.95 [ 0.937 , 0.963 ] |
| Random Forest | > 2 Models Variables | Down | 0.951 [ 0.938 , 0.964 ] |
| Random Forest | > 2 Models Variables | Simple | 0.948 [ 0.935 , 0.961 ] |
| Random Forest | > 2 Models Variables | Up | 0.949 [ 0.937 , 0.962 ] |
| Random Forest | > 1 Model Variables | Down | 0.95 [ 0.937 , 0.962 ] |
| Random Forest | > 1 Model Variables | Simple | 0.95 [ 0.937 , 0.963 ] |
| Random Forest | > 1 Model Variables | Up | 0.954 [ 0.941 , 0.966 ] |
| Random Forest | ANN Variables | Down | 0.943 [ 0.93 , 0.956 ] |
| Random Forest | ANN Variables | Simple | 0.945 [ 0.932 , 0.958 ] |
| Random Forest | ANN Variables | Up | 0.948 [ 0.935 , 0.961 ] |
| Random Forest | Random Forest Variables | Down | 0.953 [ 0.94 , 0.965 ] |
| Random Forest | Random Forest Variables | Simple | 0.954 [ 0.942 , 0.967 ] |
| Random Forest | Random Forest Variables | Up | 0.957 [ 0.943 , 0.969 ] |
| Random Forest | RBF SVM Variables | Down | 0.939 [ 0.926 , 0.952 ] |
| Random Forest | RBF SVM Variables | Simple | 0.935 [ 0.922 , 0.947 ] |
| Random Forest | RBF SVM Variables | Up | 0.936 [ 0.923 , 0.949 ] |
|  |  |  |  |
| RBF SVM | All Variables | Down | 0.93 [ 0.918 , 0.943 ] |
| RBF SVM | All Variables | Simple | 0.932 [ 0.919 , 0.945 ] |
| RBF SVM | All Variables | Up | 0.929 [ 0.916 , 0.942 ] |
| RBF SVM | Penalised LR Variables | Down | 0.951 [ 0.938 , 0.964 ] |
| RBF SVM | Penalised LR Variables | Simple | 0.951 [ 0.938 , 0.964 ] |
| RBF SVM | Penalised LR Variables | Up | 0.949 [ 0.936 , 0.961 ] |
| RBF SVM | > 2 Models Variables | Down | 0.95 [ 0.938 , 0.963 ] |
| RBF SVM | > 2 Models Variables | Simple | 0.95 [ 0.936 , 0.962 ] |
| RBF SVM | > 2 Models Variables | Up | 0.952 [ 0.939 , 0.964 ] |
| RBF SVM | > 1 Model Variables | Down | 0.945 [ 0.932 , 0.958 ] |
| RBF SVM | > 1 Model Variables | Simple | 0.94 [ 0.927 , 0.952 ] |
| RBF SVM | > 1 Model Variables | Up | 0.943 [ 0.93 , 0.955 ] |
| RBF SVM | ANN Variables | Down | 0.938 [ 0.925 , 0.951 ] |
| RBF SVM | ANN Variables | Simple | 0.941 [ 0.928 , 0.954 ] |
| RBF SVM | ANN Variables | Up | 0.94 [ 0.927 , 0.953 ] |
| RBF SVM | Random Forest Variables | Down | 0.954 [ 0.941 , 0.967 ] |
| RBF SVM | Random Forest Variables | Simple | 0.961 [ 0.947 , 0.973 ] |
| RBF SVM | Random Forest Variables | Up | 0.959 [ 0.947 , 0.972 ] |
| RBF SVM | RBF SVM Variables | Down | 0.934 [ 0.921 , 0.947 ] |
| RBF SVM | RBF SVM Variables | Simple | 0.933 [ 0.92 , 0.946 ] |
| RBF SVM | RBF SVM Variables | Up | 0.931 [ 0.919 , 0.944 ] |

**Supplementary Table 5 Caption**: *Classification performance (AUROC) for each of the different machine learning techniques using all variables and resampling approaches during model training: Simple = No resampling; Down = Randomly downsampling individuals with ND-GCs to be equal in number to controls; Up Randomly upsampling controls to be equal in number to individuals with ND-GCs. ANN: Artificial Neural Network; LR: Logistic Regression; SVM: Radial Basis Function Support Vector Machine*

## Supplementary Table 6

| **Covariate** | **Covariate Value** | **Brier Score** | **AUROC** |
| --- | --- | --- | --- |
| Age | (9.26,12.2] | 0.113 | 0.897 |
|  | (12.2,21.6] | 0.034 | 0.989 |
|  | (7.27,9.26] | 0.085 | 1 |
|  | [5.89,7.27] | 0.244 | 0.841 |
| Gender | Male | 0.109 | 0.933 |
|  | Female | 0.126 | 0.876 |

**Supplementary Table 6**: *Performance statistics for the Random Forest model fit to the 30 top variables identified by a Random Forest model fit to all variables, split by age and gender*

## Supplementary Table 7

| Variable | Variable Definition | Variable Type | Dimension Name |
| --- | --- | --- | --- |
| CNS | Considerate of other people's feelings  0 - not true  1 - somewhat true  2 - certainly true | ordinal | 1: Conduct |
| HRT | Helpful if someone is hurt  0 - not true  1 - somewhat true  2 - certainly true | ordinal | 1: Conduct |
| INC | Inconsiderate of others  0 - not true  1 - somewhat true  2 - certainly true | ordinal | 1: Conduct |
| BLT | Do they tend to blurt out the answers before the person's finished asking the question?  0 - no  1 - yes | binary | 1: Conduct |
| ALO | Do they try to avoid being on their own?  0 - no  1 - yes | binary | 2: Separation Anxiety |
| ANT | Are they distressed when they think you might be going to leave them? Or when they have to leave you?  0 - no  1 - yes | binary | 2: Separation Anxiety |
| SIT | Situational Anxious Affect  0 - no  1 - yes | binary | 3: Situational Anxiety/Sleep |
| AGO | Agoraphobia  0 - no  1 - yes | binary | 3: Situational Anxiety/Sleep |
| INI | Is it hard for them to fall asleep when they want to?  0 - no  1 - yes | binary | 3: Situational Anxiety/Sleep |
| PIM | When they were 4 to 5 did they ever play imaginative games with another child in such a way that you could tell they understood what each other was pretending?  0 - yes  1 - no | binary | 4: Communication/Play |
| REA | Is your child behind in reading?  0 - no  1- yes | binary | 4: Communication/Play |
| EST | Does your child have and educational health care plan?  0 - no  1 - yes | binary | 4: Communication/Play |
| SP2 | Was your child talking by the age of two?  0 - no  1 - yes | binary | 4: Communication/Play |
| SLT | Has your child had speech therapy?  0 - no  1 - no | binary | 4: Communication/Play |
| PCO | When they were 4 to 5 did they play co-operatively in games that need some form of joining in with a group of other children, such as hide and seek or ball games?  0 - yes  1 - no | binary | 4: Communication/Play |
| CRF | Runs as fast and easily as other children  1 - extremely like your child  2 - quite a bit like your child  3 - moderately like your child  4 - a bit like your child  5 - not at all like your child | ordinal | 5: Movement/Co-ordination |
| COB | Can organise her body to do a planned motor activity  1 - extremely like your child  2 - quite a bit like your child  3 - moderately like your child  4 - a bit like your child  5 - not at all like your child | ordinal | 5: Movement/Co-ordination |
| CGM | Likes participating in games requiring good motor skills  1 - extremely like your child  2 - quite a bit like your child  3 - moderately like your child  4 - a bit like your child  5 - not at all like your child | ordinal | 5: Movement/Co-ordination |
| CBA | Catches a small ball thrown from 6 - 8ft  1 - extremely like your child  2 - quite a bit like your child  3 - moderately like your child  4 - a bit like your child  5 - not at all like your child | ordinal | 5: Movement/Co-ordination |

**Supplementary Table 7 Caption**: *Final variables and associated dimensions identified using bootstrap exploratory graph analysis.*

# Supplementary Figure 1


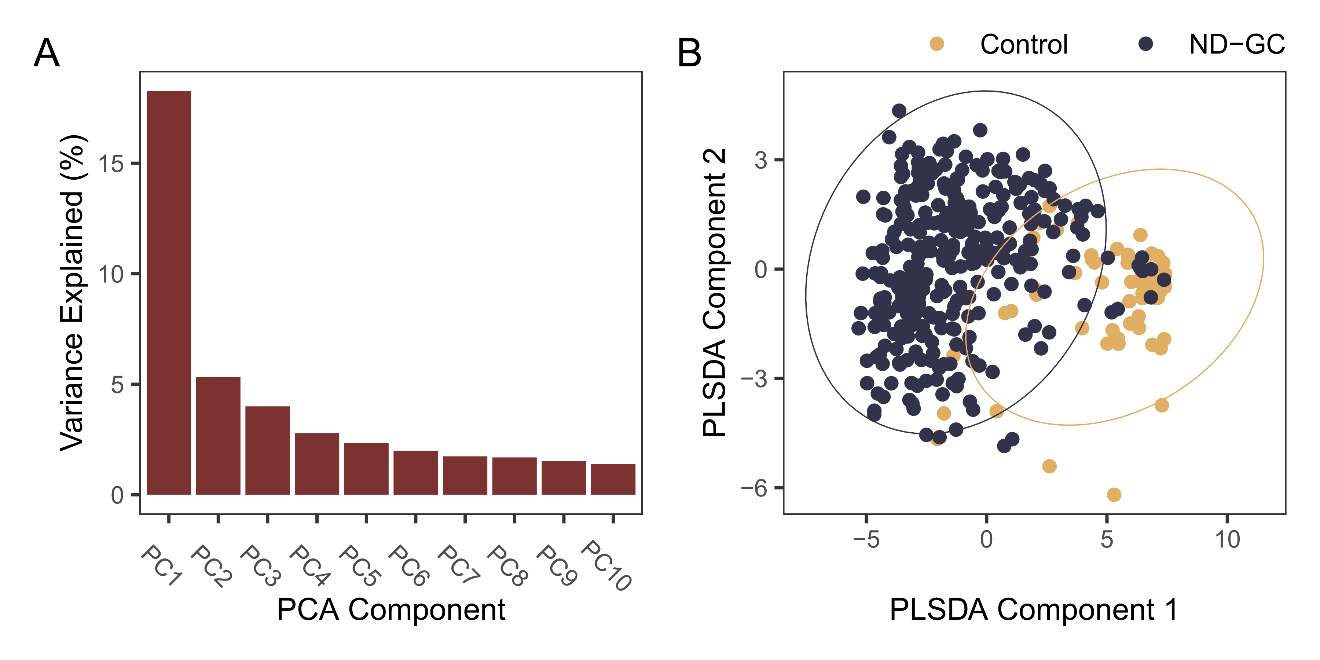


**Supplementary Figure 1 Caption**: *PCA and PLSDA. A: Variance explained by the first 10 principal components of 176 variables in 393 participants in the training dataset. One component explains a particularly large proportion of variance (18.2%). B: scatter plot of all participants by the first two PLSA components, with 95% confidence ellipse for each class.*

# Supplementary References

1. Rohart F, Gautier B, Singh A, Lê Cao KA. mixOmics: An R package for ’omics feature selection and multiple data integration. PLoS Comput Biol. 2017 Nov;13(11):e1005752.

2. Carpenter B, Gelman A, Hoffman MD, Lee D, Goodrich B, Betancourt M, et al. Stan: A Probabilistic Programming Language. J Stat Softw. 2017 Jan 11;76(1):1–32.

3. Makowski D, Ben-Shachar MS, Chen SHA, Lüdecke D. Indices of Effect Existence and Significance in the Bayesian Framework. Front Psychol [Internet]. 2019 [cited 2021 Apr 15];10. Available from: https://www.frontiersin.org/articles/10.3389/fpsyg.2019.02767/full
